# Rapid reversal of a potentially constraining genetic covariance between leaf and flower traits in *Silene latifolia*

**DOI:** 10.1002/ece3.5932

**Published:** 2019-12-16

**Authors:** Janet C. Steven, Ingrid A. Anderson, Edmund D. Brodie, Lynda F. Delph

**Affiliations:** ^1^ Department of Biology Indiana University Bloomington Indiana; ^2^ Present address: Department of Organismal and Environmental Biology Christopher Newport University Newport News Virginia; ^3^ Present address: Vanderbilt‐Ingram Cancer Center Vanderbilt University School of Medicine Nashville Tennessee; ^4^ Present address: Mountain Lake Biological Station and Department of Biology University of Virginia Charlottesville Virginia

**Keywords:** artificial selection, genetic constraint, genetic correlation, genetic variance–covariance matrix, *Silene latifolia*, specific leaf area

## Abstract

Genetic covariance between two traits generates correlated responses to selection, and may either enhance or constrain adaptation. *Silene latifolia* exhibits potentially constraining genetic covariance between specific leaf area (SLA) and flower number in males. Flower number is likely to increase via fecundity selection but the correlated increase in SLA increases mortality, and SLA is under selection to decrease in dry habitats. We selected on trait combinations in two selection lines for four generations to test whether genetic covariance could be reduced without significantly altering trait means. In one selection line, the genetic covariance changed sign and eigenstructure changed significantly, while in the other selection line eigenstructure remained similar to the control line. Changes in genetic variance–covariance structure are therefore possible without the introduction of new alleles, and the responses we observed suggest that founder effects and changes in frequency of alleles of major effect may be acting to produce the changes.

## Graphical abstract

Several studies in *Silene latifolia* support the presence of a stable, potentially constraining genetic covariance between specific leaf area and flower number. However, we were able to reverse this covariance entirely in four generations of experimental selection. Covariances among traits may be more changeable than previously suspected.

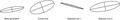

## 1 INTRODUCTION

The outcome of natural selection on groups of traits is dependent on both the strength and direction of selection and the genetic covariance between traits (Lande & Arnold, 1983). Although genetic covariances are often viewed as constraints to adaptive evolution (Arnold, 1992; Cheverud, 1984; Maynard Smith et al., 1985), their role in evolutionary dynamics can be more complex. Genetic covariances tend to orient in the direction of bivariate selection, are likely to enhance adaptive evolution, and may play an important role in promoting modularity (Agrawal & Stinchcombe, 2009; Wagner, Pavlicev, & Cheverud, 2007). Some studies have demonstrated stability in covariances over long periods of time (Arnold, Bürger, Hohenlohe, Ajie, & Jones, 2008; Bégin & Roff, 2003), while both theoretical and empirical studies suggest that covariances have the potential to change significantly in a small number of generations (Björklund, Husby, & Gustafsson, 2013; Bohren, Hill, & Robertson, 1966; Bradshaw, Emerson, & Holzapfel, 2012; Delph, Steven, Anderson, Herlihy, & Brodie, 2011). At this time, it is unclear the extent to which the stability of genetic covariance slows adaptive evolution in lineages.

In this study, we attempted to disrupt a stable and potentially constraining positive covariance between flower number and leaf thinness, as measured by specific leaf area (SLA), in males of the plant species *Silene latifolia*. This species is dioecious and sexually dimorphic for both traits, with males producing more flowers and thinner leaves than females (Delph & Bell, 2008; Delph, Knapczyk, & Taylor, 2002). Although the covariance is positive, it represents a potential constraint to the evolution of traits toward their optima in male plants. Increased flower number contributes to higher fecundity, but the correlated increase in SLA means plants also make thinner leaves, which are associated with higher transpiration rates and increased mortality (Delph & Herlihy, 2011). Selection for decreased SLA in males is significant in dry habitats (Delph, Andicoechea, et al., 2011).

Despite the conflict between SLA and flower number in *S. latifolia*, a positive and significant correlation between them has been observed in several studies. A phenotypic correlation between flower number and SLA was seen across nine populations in the United States and Europe (Delph et al., 2002), and in a different population the estimated genetic correlation between SLA and flower number was positive and significant (Delph, Andicoechea, et al., 2011). A selection experiment on flower size generated correlated change in both flower number and SLA, suggesting genetic integration of these traits (Delph, Gehring, Arntz, Levri, & Frey, 2005). Lastly, a quantitative trait locus (QTL) for flower number was found to overlap with a QTL for SLA, indicating possible linkage or pleiotropy between the traits (Delph, Arntz, Scotti‐Saintagne, & Scotti, 2010). In general, a positive genetic correlation between SLA and flower number appears to be pervasive and stable, and may constrain the evolution of each trait toward its optimum value. In other words, there may be environments in which making relatively many flowers and having thick leaves would be optimal for male plants via both fecundity and viability selection, but this combination of traits opposes the observed correlation. 

We anticipate that a change in the covariance between flower number and SLA in male *S. latifolia* is possible through changes in existing genetic variation and can therefore occur in a small number of generations. Covariances are potentially shaped by linkage disequilibrium, pleiotropy, and differential epistasis and are influenced by the relative contributions of these mechanisms as well as the rate of changes in allele frequency and loss of alleles caused by fixation. Loci in linkage disequilibrium can generate a correlation between two traits that is susceptible to rapid change (Falconer & Mackay, 1996). While selection favors linkage when covariance is adaptive (Sinervo & Svensson, 2002), covariance generated by linkage disequilibrium is expected to decrease over time in the absence of selection. In addition, fixation resulting from selection or drift at one or both loci eliminates the influence of the linkage on covariance, reducing its magnitude even if linkage is not disrupted. Pleiotropy has the potential to generate stable, lasting genetic covariation (Mitchell‐Olds, 1996). However, a small number of pleiotropic alleles and/or alleles with variable or opposing pleiotropic effects can reduce the stability of covariation, such that changes in allele frequency and loss of alleles could significantly alter the magnitude of covariation in a small number of generations (Agrawal, Brodie, & Rieseberg, 2001; Bohren et al., 1966; Conner, 2012; Falconer & Mackay, 1996; Gromko, 1995; Houle, 1991). Differential epistasis occurs when the extent of pleiotropy caused by a locus is modified by alleles at a second locus (Cheverud, 2001) and may also contribute to a rapid change in covariance. Moreover, changes at a locus influencing differential epistasis have the potential to modify genetic covariances without changing trait means (Pavlicev et al., 2008). In sum, several genetic mechanisms have the potential to cause a rapid change in genetic covariance, suggesting that shifts away from a constraining correlation are possible under certain conditions and selection regimes.

The time scale over which genetic covariance can change has implications for the ability of organisms to escape developmental or functional constraints and adapt to new environments. To determine whether a genetic covariance could change rapidly while trait means changed little, we conducted an artificial‐selection experiment in *S. latifolia* in which we selected families with combinations of trait values for flower number and SLA that had the potential to reduce the covariance but keep trait means constant.

## 2 MATERIALS AND METHODS

### 2.1 Study species

*Silene latifolia* (Caryophyllaceae) is a short‐lived perennial herb native to Europe and widely common as an introduced species in the United States. It is dioecious and sexually dimorphic for many reproductive and vegetative traits (see review in Delph, 2007). It flowers in its first year, begins flowering in mid‐spring or early summer, and produces flowers indeterminately on a cyme (Morton, 2005). Its white, fragrant flowers open at night and are primarily pollinated by moths (Shykoff & Bucheli, 1995). Fruits are capsules containing many small seeds.

### 2.2 Trait measurements and selection procedures

**Figure 1:**
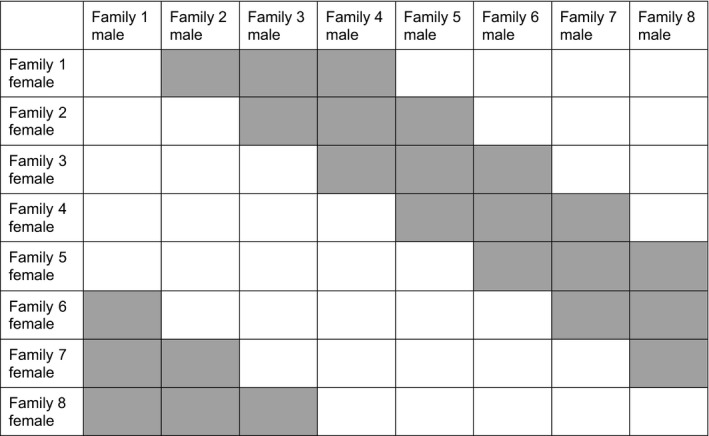
Diagram of the crossing design used in selection and control lines. Gray cells indicate crosses that were performed

The base generation for selection was founded from seeds collected in a wild population in Giles County, Virginia. Seeds from 103 capsules were grown in a greenhouse at Indiana University (IU). These plants were used as parents in a breeding design that used a male and a female originating from each capsule and mated each individual to three other individuals to generate 150 full‐sib families. Each family had two other families with the same mother and two other families with the same father, generating additional half‐sib relationships. For example, a female from family 1 was mated to males from families 2, 3, and 4, and a female from family 2 was mated to males from families 3, 4, and 5 (Figure 1). See Steven, Delph, and Brodie (2007) for further details of the crossing design. A randomly selected subset of 120 of the resulting full‐sib families was used in this study.

We grew all plants in this study in the same environment in pollinator‐free greenhouses at IU, and we used a mix of Metromix (Scotts‐Sierra Horticultural Products) and sterilized field soil for seed germination and transplanting. Seeds were first germinated in celled trays, and then transplanted into 5″ clay pots. To reduce the effects of variation in greenhouse conditions, we moved plants among benches weekly. We used a single treatment of imidacloprid pesticide (Marathon brand; OHP Inc.) before flowering began, and plants received half‐strength 20:20:20 Peter's fertilizer (Scotts‐Sierra Horticultural Products) every 10 days.

We measured specific leaf area (SLA) 10 days after a plant opened its first flower on the leaf two nodes down from that flower. If the leaf at the second node was smaller than 3 cm^2^, we used the third leaf down. Leaf area was measured with a portable leaf‐area meter (Li3000‐A; Li‐Cor), and leaves were then dried in a drying oven to constant weight and weighed. We calculated SLA as area in cm^2^ per g of leaf tissue. Flower number was counted 30 days after the plant opened its first flower. All pedicels with missing flowers, open flowers, and flowers preparing to open that night were counted. In the base generation, we measured specific leaf area and flower number on a total of 262 male plants. An average of 2.3 plants were measured per full‐sib family, and seven families produced no males.

**Figure 2:**
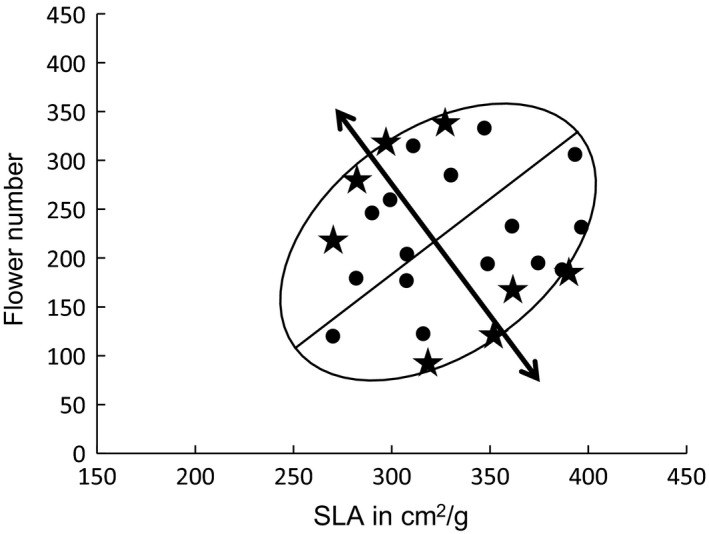
Diagram of our selection procedure. Ellipse represents the magnitude and orientation of the first and second principal components. Points and stars represent mean values for each of 24 families. Stars denote the families with the most extreme values in both directions on the second principal component, which would be selected for the next generation

Selection to directly alter the correlation was similar to the selection employed in Delph, Steven, et al. (2011). From the base generation, we established a control line and two replicate selection lines by selecting eight full‐sib families for each. The goal of selection was to decrease the covariance between male SLA and male flower number while keeping the trait means the same. In other words, we performed disruptive correlational selection on the two traits. To achieve this, we identified families with trait mean combinations most distant from the major axis of the correlation using principal components analysis. We used the second principal component value (PC2) to identify families for selection (Figure 2). In an attempt to maintain trait means, we selected full‐sib families equally from both extremes of the second axis; four families with the most positive PC2 values, and four with the most negative. In the first round of selection from the base generation, we attempted to maintain similar selection strength in the two selection lines (Table 1). Families for the control line were selected randomly, and we allowed a family to be used in both the control line and a selection line to avoid bias.

**Table 1:** Disruptive correlational selection differentials and directional selection differentials on the principal components for the control and selection lines, and number of males measured

Line	Generation	Disruptive correlational selection differential for PC2	Directional selection differential for PC1	Directional selection differential for PC2	Number of individuals
Control	Base	−0.12	0.24	−0.05	265
	1	−0.05	0.25	−0.23	69
	2	0.03	−0.18	0.19	60
	3	−0.22	0.06	−0.12	38
Selection 1	Base	0.74	0.24	−0.08	265
	1	0.53	0.14	−0.02	66
	2	0.57	0.23	0.04	52
	3	0.66	0.31	−0.05	53
Selection 2	Base	0.83	0.10	−0.08	265
	1	0.60	−0.45	−0.07	54
	2	0.69	0.05	−0.01	67
	3	0.47	0.27	−0.04	42

Note

Disruptive correlational selection differentials were calculated from the deviation of each selected family along the second principal component. The directional selection differentials are a byproduct of this selection. Twenty‐four families were created for each generation, and eight families were selected to act as parents in the next generation.

To select parents from each full‐sib family for breeding, we randomly selected a female and chose the male with trait values most similar to the family mean. In some cases, selection of the male was determined by availability of flowers and pollen. We performed crosses using the same general design used to create the base generation (Figure 1). This design generated 24 full‐sib families. When performing selection for the next generation, we selected from these full‐sib families.

We selected to reduce the correlation between SLA and flower number in males over a total of four generations. In generations 1–3, we grew eight seeds per family under the same conditions as the base generation and followed the same selection and crossing procedures. Sample sizes ranged from 38 to 69 males measured per line.

For the fourth and final generation, we grew a larger number of seeds from each of the 24 full‐sib families to generate a sample size adequate for estimating genetic covariance. All lines were grown together under the same environmental conditions, and traits were measured using the same protocols as the earlier generations, with the exception that plants were fertilized monthly. Both traits were measured on 218 plants in the control line, 230 plants in selection line 1, and 148 plants in selection line 2. An average of 8.9 plants per family was measured, with an average relatedness of 0.25 (Table 2).

**Table 2:** Number of pairwise full‐ and half‐sib relationships and average pairwise relatedness in the control line and two selection lines

	Full sib	Maternal half sib	Paternal half sib	Average relatedness
Control line	1,269	3,694	3,758	0.264
Selection line 1	1,490	4,102	4,127	0.250
Selection line 2	997	2,780	2,321	0.239

Note

Pairwise relationships are determined for the final generation, and average relatedness is calculated for the full pedigree.

### 2.3 Statistical analysis

Disruptive correlational selection differentials were calculated by taking the average absolute value of the second principal component for only the families selected for the next generation and subtracting the average absolute value of the principal component for every family in the line. We also calculated directional selection differentials for the first and second principal components to determine possible inadvertent selection on the means.

The number of pairwise full and half sib relationships in the last generation of the control line and the two selection lines were calculated using the R package “pedantics” (Morrissey & Wilson, 2010). We also used this package to calculate average relatedness for the full pedigree in each line.

To determine whether selection changed mean trait values, we used a one‐way analysis of variance with line as the factor for each trait. We compared means between lines using Tukey's HSD.

We estimated genetic parameters for the base generation and each of the lines in generation four using the multivariate animal model (Kruuk, 2004; Wilson et al., 2010) and restricted maximum likelihood in ASReml‐R 3.3 software (VSN International Ltd; http://www.vsni.co.uk). Flower number was log‐transformed and multiplied by 50 prior to analysis to make the variable normally distributed and to scale it similarly to specific leaf area. Because the unit of observation in the animal model is the individual and not the family, ASReml calculates a relationship matrix between individuals that includes all information about full‐sib, half‐sib, and cousin relationships. We conducted separate analyses for the base generation and the last generation of each line, and within each we included the relevant pedigree information from all generations of breeding. The model estimated mean trait values as a fixed effect and additive genetic variance–covariance values as a random effect. Preliminary analysis showed no significant maternal effects, and we did not include them in the final model. To test whether genetic covariances were significantly greater or less than 0, we used log‐likelihood ratio tests that compared the full model to a model that constrained genetic covariance to 0. We used ASReml‐R to estimate standard errors for genetic variance and covariance values and to estimate heritabilities for SLA and flower number and standard errors for those values.

To visualize the differences in overall G matrix structure among lines, we generated an ellipse representing the eigenstructure of the matrix for the base generation and the last generation of each line. Using eigenvalues, we calculated major and minor axes scaled to 95% of the variation; these values determine the x and y dimensions of the ellipse. The angle of the ellipse was calculated from the eigenvectors and captures the rotation that would be imposed by the matrix on a selection gradient.

To statistically compare the eigenstructure of the selection lines to the eigenstructure of the base generation and the control line, we generated null distributions with the hypothesis that the two populations being compared did not differ in genetic structure. For each comparison, we generated simulated populations by selecting a random sample of 100 individuals from each of the two populations and combining them into a single dataset. We then used the ASReml model described above to estimate the G matrix for the dataset. From this matrix, we used eigenvalues and eigenvectors to calculate the major axis, minor axis, and angle of rotation. We repeated this process 1,000 times to generate null distributions for each parameter, then compared the actual value from each selection line to the appropriate null distribution. If the null hypothesis of similarity is true, then the observed values from the selection line should fall within the null distribution generated from combined populations. We calculated the *p*‐value for each comparison from the number of randomly generated populations with values more extreme than the observed value.

We also used random skewers to compare G matrices among lines (Cheverud & Marroig, 2007). The random skewers test compares matrices within the context of the multivariate breeder's equation. Random selection vectors are multiplied by each variance–covariance matrix, and the similarity of the response vectors is determined by the vector correlation between them. If two matrices result in similar responses to the same selection vectors, the correlation is high. To determine statistical significance, this correlation is compared against a null distribution of vector correlations between vectors selected randomly from a uniform distribution. Therefore, a *p*‐value <.05 indicates greater matrix similarity than expected by chance. We used the R package phytools 0.5–20 (Revell, 2012) to conduct pairwise comparisons between the genetic variance–covariance matrices for the base generation and the control and selection lines.

## 3 RESULTS

**Figure 3:**
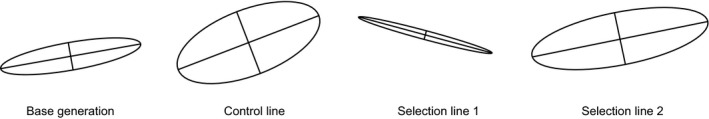
Ellipses representing the eigenstructure of the G matrix in the base generation, the control line after four generations of random mating, and selection line 1 and selection line 2 after four generations of selection to reduce the covariance between the traits. The magnitude of the major and minor axes correspond to eigenvalues, and the angle of the matrix represents matrix rotation, as determined by eigenvectors

After four generations of selection, the genetic covariance in selection line 1 was negative and significantly <0 (*χ* ^2^ = 4.58, *p* = .032; Table 3). The eigenstructure of the G matrix in selection line 1 also deviated significantly from the base generation and the control line (Figure 3, Table 4). However, the eigenstructure of the G matrix in selection line 2 was similar to the control line, and the genetic covariance was not significantly different from 0 (*χ* ^2^ = 0.819, *p* = .37). In both selection line 2 and the control line, genetic variation increased somewhat (Table 3), and the genetic covariance in the control line was also not significantly different from 0 (*χ* ^2^ = 2.18, *p* = .140).

**Table 3:** Additive genetic variance and covariance for flower number (log‐transformed and multiplied by 50) and specific leaf area (cm^2^/g) in males for plants in the base generation, the control line after four generations of random mating, and selection line 1 and selection line 2 after four generations of selection to reduce the correlation between the traits

	*V*_A_				
	Flower number	SLA	COV_A_	*r*_P_	*r*_G_
Base generation	47 ± 28	697 ± 229	116 ± 58*	0.08	0.64
Control line	233 ± 127	720 ± 340	212 ± 162	0.35	0.52
Selection line 1	52 ± 55	617 ± 298	−174 ± 98*	0.07	−0.96
Selection line 2	128 ± 84	1,093 ± 597	177 ± 174	0.30	0.47

Note

Values are followed by ±1 *SE*. Covariances marked * are significantly different from 0 at *p* < .05 based on a log‐likelihood ratio test. Correlations are presented as standardizations of covariance and no significance testing was conducted for them.

**Table 4:** Comparison of G matrix structure among lines

	Major axis		Minor axis		Angle of rotation	
	Base	Control	Base	Control	Base	Control
Selection line 1	*p* < .001	*p < *.001	*p = *.023	*p = *.003	*p* < .001	*p* < .001
Selection line 2	n.s.	n.s.	n.s.	n.s.	n.s.	n.s.

Note

To determine whether the lines deviated from the base generation, and whether the selection lines differed significantly from the control line, we conducted randomization tests that generated a null distribution of 1,000 iterations from a combined dataset, and *p*‐values were calculated from the number of iterations that were more extreme than the observed values.

The genetic correlations calculated from the variance and covariance estimates reflect the changes in both variance and covariance. In the control line and selection line 2, where variance increased for both traits, the genetic correlation between traits was slightly smaller than the genetic correlation in the base generation (Table 3). In selection line 1, the reduction in genetic variance in both traits and the reversal in sign of the genetic covariance resulted in a highly negative genetic correlation between traits. Phenotypic correlations were generally small and not similar to the underlying genetic correlations (Table 3).

**Figure 4:**
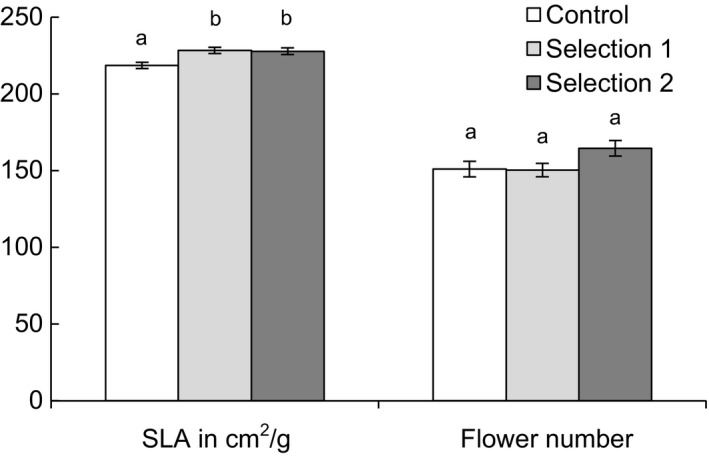
Mean specific leaf area (SLA) and mean flower number 30 days after flowering began for male *Silene latifolia* in a control line mated randomly for four generations and two selection lines in which selection to disrupt the correlation between flower number and specific leaf area was imposed. Between 148 and 230 males were counted per line. Means within a trait that share a letter are not significantly different, based on an ANOVA and Tukey's HSD (SLA: *F*_2,681_ = 7.18, *p* = .001, flower number: *F*_2,593_ = 2.26, *p* = .106)

Selection to alter the covariance had weak effects on mean trait values in both selection lines. Trait means for the two selection lines in the final generation only deviated slightly from the control line (Figure 4). Specific leaf area was greater in the two selection lines than the control, but only by 4%. In both selection lines, mean flower number was not significantly different from the control. Overall, selection on the covariance had at most a slight effect on trait means.

**Figure 5:**
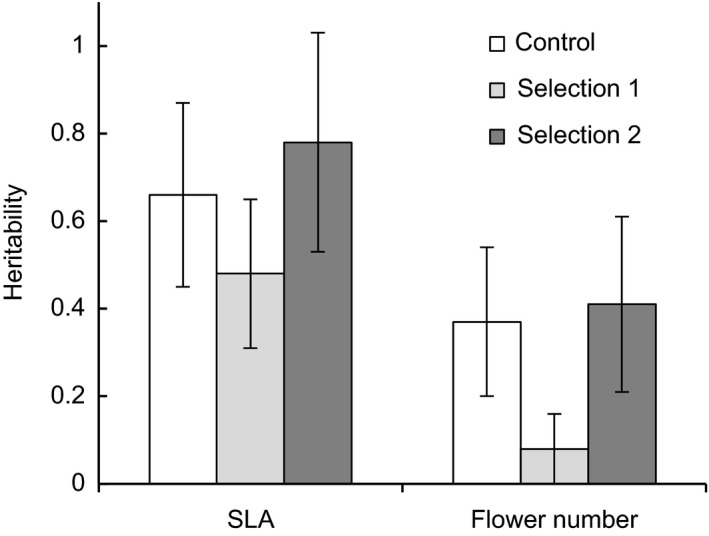
Heritability of specific leaf area (SLA) and flower number in male *Silene latifolia* after four generations of selection to break the correlation between the traits. Error bars are ±1 *SE*

The heritabilities of SLA and flower number in males were somewhat affected by selection (Figure 5). After four generations, the heritability of flower number in selection line 1 had decreased. In addition, the additive genetic variance for flower number in selection line 1 remained similar to the genetic variance present in the base generation while it increased in both the control line and selection line 2 (Table 2). Selection line 2 also showed an appreciable increase in additive genetic variance for SLA (Table 2).

The differing responses in the two selection lines were also evident in the eigenstructure of the G matrix after selection. The eigenstructure of selection line 1 diverged significantly from the eigenstructure of the base generation and the control line, reflecting a significant change in genetic variation and covariation in this line (Table 4). The rotation of the matrix increased above 90 degrees, indicating a negative correlation between flower number and specific leaf area. In addition, the magnitude of variation along the minor axis decreased considerably (Figure 3). However, selection line 2 did not deviate significantly in eigenstructure from either the base generation or control line (Figure 3, Table 4).

Matrix comparison by random skewers also revealed that the G matrix of selection line 1 was altered significantly compared with that of the base generation and the control line (Table 5). The G matrix of selection line 2 differed significantly from that of the control, although correlations among all matrices were generally high.

**Table 5:** Comparison of genetic variance–covariance matrices by random skewers

	Control line	Selection line 1	Selection line 2
Base generation	0.912	0.904	0.992*
Control line		0.791	0.949
Selection line 1			0.894

Note

Values are vector correlations between response vectors for the two matrices; higher correlations indicate greater similarity in responses to random selection vectors. The asterisk indicates the correlation that showed significant similarity between the two matrices.

## 4 DISCUSSION

The selection regime we imposed effectively altered the genetic covariance between SLA and flower number in males of *S. latifolia* in one of the two selection lines. In addition, the genetic variance–covariance structure of the control line shifted under random mating. These rapid changes in the structure of the G matrix were derived from existing genetic (co)variation, suggesting that the genetic architecture that underlies these two traits is labile even in the absence of new allelic variation introduced through mutation or gene flow. The differing responses between the two replicate selection lines also point to the importance of stochastic factors in shaping changes in genetic architecture over generations. The reversal of the genetic covariance in selection line 1 within 4 generations suggests that standing allelic variation at one or very few loci can lead to qualitative changes in the structure of G matrices.

The covariance in selection line 1, while opposite in sign, is of similar magnitude to the covariance in the control and selection lines. However, the associated genetic correlation is very strong. This large negative genetic correlation in line 1 is potentially influenced by the reduction in genetic variation for both traits. While selection expanded genetic variance for flower number in selection line 2, genetic variance and heritability for flower number in selection line 1 was low in comparison. This small genetic variance essentially shrinks the denominator in the formula for the correlation, and most of the genetic control over flower number is then determined by the covariance that appears in the numerator. This phenomenon has potentially produced a very tight genetic correlation between the two traits but reduced genetic variance for the traits in general.

While both the magnitude and orientation of the G matrix in selection line 1 was altered by our selection regime, the eigenstructure of the G matrix was generally similar across the base generation, the control line, and the second selection line. In addition, the random skewers test provided evidence that the overall effect of the reduced covariance on the outcome of selection is surprisingly modest. When multiplied by random selection vectors, the G matrix for selection line 1 generated a response to selection correlated with the response in the other selection line and the control line, indicating that the matrices retain some similarity after selection. However, the greatest difference in matrices was between selection line 1 and the control, reflecting the significant change in covariance in this line.

The magnitude and rapidity of the change in the genetic covariance in selection line 1 suggest that one or a few loci of major effect may be driving the switch in sign. Other studies have demonstrated that single alleles with pleiotropic effects can significantly alter a correlation over a few generations (Agrawal et al., 2001). Segregating alleles at a single locus in *Arabidopsis thaliana* had effects on the value of a genetic correlation (Stinchcombe, Weinig, Heath, Brock, & Schmitt, 2009), and an increased mutation rate significantly altered the covariance structure of size and reproductive investment traits in *A. thaliana*, while trait means did not change (Camara & Pigliucci, 1999). In addition, strong selection for an insecticide resistance allele in a leafroller led to changes in diapause and larval weight, suggesting a constraining pleiotropic effect (Carriere & Roff, 1995).

The divergent results between our two selection lines, and the change in the control line in comparison with the base generation, may result from founder effects in the initial establishment of lines, genetic drift, and fixation of alleles during the selection process. If a few alleles with significant pleiotropic effects are influencing the covariance, chance increases or decreases in their frequency in the populations that founded the lines, or potentially loss of alleles during the founding process, could influence the outcome of selection and random mating. Fixation or loss of pleiotropic alleles in both the selection and control lines during the experiment could alter the covariance because loci fixed at one allele no longer contribute to genetic variance and covariance; the fixation of pleiotropic alleles in selection line 1 may also be responsible for the decrease in genetic variance for each trait. Fixation of an allele controlling differential epistasis in selection line 1 could have also contributed to the change in covariance. Additional replicate selection lines would be informative in determining how much stochastic factors influence the outcome of selection on a covariance, and whether our two selection lines represent the extremes of a range of possible outcomes.

**Figure 6:**
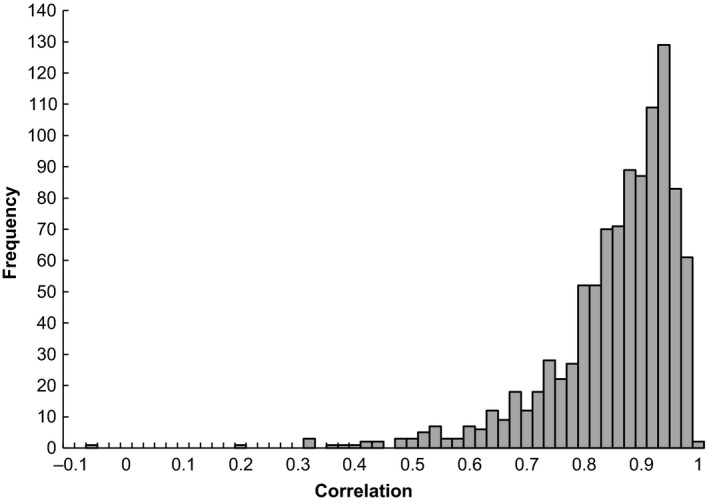
Histogram of simulated genetic correlations calculated from best linear unbiased predictors for specific leaf area and flower number randomly sampled from 12 individuals in the base generation. Resampling was conducted 1,000 times

To determine whether founder effects in the initial establishment of lines could lead to a small or negative covariance, we conducted a simulation in which we randomly selected 12 individuals from the base generation and calculated the correlation between the Best Linear Unbiased Predictors (BLUPs) for specific leaf area and flower number from the ASReml analysis. BLUPs give an individual‐level estimate of the effect of genotype on trait values. BLUPs are not direct estimates of breeding values and generate anticonservative estimates of genetic parameters (Hadfield, Wilson, Garant, Sheldon, & Kruuk, 2010). However, calculating correlations between BLUPs that overestimate the genetic correlation will decrease rather than increase our ability to detect small correlations, and here BLUPs provide a glimpse into the possible genetic composition of the founding populations of the lines. Although founding populations consisted of 16 individuals, breeding among relatives reduces effective population size. Therefore, we chose a population size of 12, which assumes 50% of the matings among the 16 individuals were between first cousins. We generated a total of 1,000 correlations. The average correlation was 0.84, which is larger than the genetic correlation of 0.62 estimated using the animal model and reflects the anticonservative nature of BLUPs. In the simulation, eighteen correlations were <0.5, and two were <0.2 (Figure 6). These findings suggest that the initial selection of individuals from the base generation had the potential to establish lines with very small initial genetic covariance between the traits and highlight the importance of stochastic factors in changes in the G matrix.

We also calculated the actual correlations between BLUPs for the founding populations in each line. The BLUPs for specific leaf area and flower number were positively correlated in the plants used to found the control line (*r* = .71, *t* = 3.82, *df* = 14, *p* = .0019), but were not significantly different from 0 in either of the selection lines (selection 1; *r* = .27, *t* = 1.054, *df* = 14, *p* = .31: selection 2; *r* = .035, *t* = 0.1316, *df* = 14, *p* = .90). The significant change in covariance in selection line 1 may have been facilitated by inclusion of alleles that did not contribute to a positive genetic correlation in the base generation.

Why then is the potentially constraining genetic correlation between SLA and flower number consistent across populations of *S. latifolia* and persistent over time, despite the fact that we were able to alter it in a few generations? Perhaps selection regimes in nature are not strong or targeted enough to generate significant change in the covariance itself. By selecting males with thicker leaves and more flowers simultaneously with males with thinner leaves and fewer flowers, we imposed a strong multivariate selection regime that would be unlikely to occur in a natural population. In addition, it is apparent from selection experiments on pairs of traits that means can change without altering the correlation (Conner et al., 2011; Frankino, Zwaan, Stern, & Brakefield, 2007), indicating that traits in wild populations may be able to reach optima despite strong genetic covariance. It is also possible that the positive covariance between the two traits is adaptive, not constraining, at least in some environments or times in the growing season. For example, in areas in which water availability is limited during the reproductive season, having both relatively thick leaves (which transpire at a lower rate than thinner leaves) and making fewer flowers may be under relatively strong viability selection, such that any selection via fertility on flower number is overwhelmed. Random skewers analysis found that the G matrix for the base generation and selection line 2 were similar, but the base generation and the control line were not, suggesting that selection may promote the structure of the matrix found in the base generation. Variability in selection pressures across a season or among years may help to maintain alleles that generate a negative or zero covariance at low frequencies by occasionally favoring these alleles (Houle, 1991).

Restructuring a covariance in a relatively small number of generations has implications both for escape from constraint and for the development of adaptive covariance between traits. Although the lability of a covariance is dependent on its underlying genetic structure, the likelihood of change depends upon optimum phenotype combinations (Arnold, 1992; Brodie, 1992). If the multivariate adaptive landscape is relatively static, the structure of the covariance may converge with the structure of the adaptive landscape even when change in covariance is slow (Arnold et al., 2008). If the covariance is capable of change that is contemporaneous with changing trait optima, the interplay between covariation and correlational selection could be a key aspect of the dynamics of adaptation even over short time scales. Selection could potentially create genetic covariance that enhances trait integration and adaptive evolution, as observed in some studies of multivariate selection (Agrawal & Stinchcombe, 2009).

## 5 CONCLUSION

Our findings illustrate the potential instability of a genetic covariance when exposed to novel selection regimes and genetic drift and suggest that variation in the underlying genetic architecture may contribute to the lability of a covariance. Changes in covariance caused by changes in allele frequency and fixation of alleles may be especially important for covariances strongly influenced by few loci of major effect. The independent evolution of correlated traits is potentially constrained not by the covariance itself, but by the combination of covariance and patterns of selection exerted by the environment.

## CONFLICT OF INTEREST

The authors declare that they have no conflict of interest.

## AUTHOR CONTRIBUTIONS

LFD and EDBIII conceived of the research question and designed the experiment; JCS and IAA collected and analyzed data. JCS drafted the manuscript and figures, which were then reviewed and revised by LFD, EDBIII, and JCS.

## Data Availability

Data files are archived in the Dryad Digital Repository at https://doi.org/10.5061/dryad.dbrv15dx7.

## References

[ece35932-bib-0001] Agrawal, A. A. , Brodie, E. D. III , & Rieseberg, L. H. (2001). Possible consequences of genes of major effect: Transient changes in the G matrix. Genetica, 112–113, 33–43. 11838774

[ece35932-bib-0002] Agrawal, A. F. , & Stinchcombe, J. R. (2009). How much do genetic covariances alter the rate of adaptation? Proceedings of the Royal Society B: Biological Sciences, 276, 1183–1191. 10.1098/rspb.2008.1671 PMC2679087 19129097

[ece35932-bib-0003] Arnold, S. J. (1992). Constraints on phenotypic evolution. American Naturalist, 140, S85–S107. 10.1086/285398 19426028

[ece35932-bib-0004] Arnold, S. J. , Bürger, R. , Hohenlohe, P. A. , Ajie, B. C. , & Jones, A. G. (2008). Understanding the evolution and stability of the G‐matrix. Evolution, 62, 2451–2461. 10.1111/j.1558-5646.2008.00472.x 18973631 PMC3229175

[ece35932-bib-0005] Bégin, M. , & Roff, D. A. (2003). The constancy of the G matrix through species divergence and the effects of quantitative genetic constraints on phenotypic evolution: A case study in crickets. Evolution, 57, 1107–1120. 10.1554/0014-3820(2003)057[1107:TCOTGM]2.0.CO;2 12836827

[ece35932-bib-0006] Björklund, M. , Husby, A. , & Gustafsson, L. (2013). Rapid and unpredictable changes of the G‐matrix in a natural bird population over 25 years. Journal of Evolutionary Biology, 26, 1–13. 10.1111/jeb.12044 23240615

[ece35932-bib-0007] Bohren, B. B. , Hill, W. G. , & Robertson, A. (1966). Some observations on asymmetrical correlated responses to selection. Genetical Research, 7, 44–57. 10.1017/S0016672300009460 5906491

[ece35932-bib-0008] Bradshaw, W. E. , Emerson, K. J. , & Holzapfel, C. M. (2012). Genetic correlations and the evolution of photoperiodic time measurement within a local population of the pitcher‐plant mosquito, *Wyeomyia smithii* . Heredity, 108, 473–479. 10.1038/hdy.2011.108 22072069 PMC3330697

[ece35932-bib-0009] Brodie, E. D. III (1992). Correlational selection for color pattern and antipredator behavior in the garter snake *Thamnophis ordinoides* . Evolution, 46, 1284–1298. 10.1111/j.1558-5646.1992.tb01124.x 28568995

[ece35932-bib-0010] Camara, M. D. , & Pigliucci, M. (1999). Mutational contributions to genetic variance‐covariance matrices: An experimental approach using induced mutations in *Arabidopsis thaliana* . Evolution, 53, 1692–1703. 10.1111/j.1558-5646.1999.tb04554.x 28565453

[ece35932-bib-0011] Carriere, Y. , & Roff, D. A. (1995). Change in genetic architecture resulting from the evolution of insecticide resistance: A theoretical and empirical analysis. Heredity, 75, 618–629. 10.1038/hdy.1995.181

[ece35932-bib-0012] Cheverud, J. M. (1984). Quantitative genetics and developmental constraints on evolution by selection. Journal of Theoretical Biology, 110, 155–171. 10.1016/S0022-5193(84)80050-8 6492829

[ece35932-bib-0013] Cheverud, J. M. (2001). The genetic architecture of pleiotropic relations and differential epistasis In Wagner G. P. (Ed.), The character concept in evolutionary biology (pp. 411–434). New York, NY: Academic Press.

[ece35932-bib-0014] Cheverud, J. M. , & Marroig, G. (2007). Comparing covariance matrices: Random skewers method compared to the common principal components model. Genetics and Molecular Biology, 30, 461–469. 10.1590/S1415-47572007000300027

[ece35932-bib-0015] Conner, J. K. (2012). Quantitative genetic approaches to evolutionary constraint: How useful? Evolution, 66, 3313–3320. 10.1111/j.1558-5646.2012.01794.x 23106699

[ece35932-bib-0016] Conner, J. K. , Karoly, K. , Stewart, C. , Koelling, V. A. , Sahli, H. F. , & Shaw, F. H. (2011). Rapid independent trait evolution despite a strong pleiotropic genetic correlation. American Naturalist, 178, 429–441. 10.1086/661907 21956022

[ece35932-bib-0017] Delph, L. F. (2007). The genetic integration of sexually dimorphic traits in the dioecious plant *Silene latifolia* In Fairbairn D. J., Blanckenhorn W. U., & Szekely T. (Eds.), Sex, size and gender roles. Evolutionary studies of sexual size dimorphism (pp. 115–123). Oxford, UK: Oxford University Press.

[ece35932-bib-0018] Delph, L. F. , Andicoechea, J. , Steven, J. C. , Herlihy, C. R. , Scarpino, S. V. , & Bell, D. L. (2011). Environment‐dependent intralocus sexual conflict in a dioecious plant. New Phytologist, 192, 542–552. 10.1111/j.1469-8137.2011.03811.x 21726233

[ece35932-bib-0019] Delph, L. F. , Arntz, A. M. , Scotti‐Saintagne, C. , & Scotti, I. (2010). The genomic architecture of sexual dimorphism in the dioecious plant *Silene latifolia* . Evolution, 64, 2873–2886. 10.1111/j.1558-5646.2010.01048.x 20550575

[ece35932-bib-0020] Delph, L. F. , & Bell, D. L. (2008). A test of the differential‐plasticity hypothesis for variation in the degree of sexual dimorphism in *Silene latifolia* . Evolutionary Ecology Research, 10, 61–75.

[ece35932-bib-0021] Delph, L. F. , Gehring, J. L. , Arntz, M. , Levri, M. , & Frey, F. M. (2005). Genetic correlations with floral display lead to sexual dimorphism in the cost of reproduction. American Naturalist, 166, S31–S41. 10.1086/444597 16224710

[ece35932-bib-0022] Delph, L. F. , & Herlihy, C. R. (2011). Sexual, fecundity, and viability selection on flower size and number in a sexually dimorphic plant. Evolution, 66, 1154–1166. 10.1111/j.1558-5646.2011.01510.x 22486695

[ece35932-bib-0023] Delph, L. F. , Knapczyk, F. N. , & Taylor, D. R. (2002). Among‐population variation and correlations in sexually dimorphic traits of *Silene latifolia* . Journal of Evolutionary Biology, 15, 1011–1020. 10.1046/j.1420-9101.2002.00467.x

[ece35932-bib-0024] Delph, L. F. , Steven, J. C. , Anderson, I. A. , Herlihy, C. R. , & Brodie, E. D. III (2011). Elimination of a genetic correlation between the sexes via artificial correlational selection. Evolution, 65, 2872–2880. 10.1111/j.1558-5646.2011.01350.x 21967428

[ece35932-bib-0025] Falconer, D. S. , & Mackay, T. F. C. (1996). Introduction to quantitative genetics (4th ed.) San Francisco, CA: Benjamin Cummings.

[ece35932-bib-0026] Frankino, W. A. , Zwaan, B. J. , Stern, D. L. , & Brakefield, P. M. (2007). Internal and external constraints in the evolution of morphological allometries in a butterfly. Evolution, 61, 2958–2970. 10.1111/j.1558-5646.2007.00249.x 17976182 PMC3198855

[ece35932-bib-0027] Gromko, M. H. (1995). Unpredictability of correlated response to selection: Pleiotropy and sampling interact. Evolution, 49, 685–693. 10.1111/j.1558-5646.1995.tb02305.x 28565144

[ece35932-bib-0028] Hadfield, J. D. , Wilson, A. J. , Garant, D. , Sheldon, B. C. , & Kruuk, L. E. B. (2010). The misuse of BLUP in ecology and evolution. American Naturalist, 175, 116–125. 10.1086/648604 19922262

[ece35932-bib-0029] Houle, D. (1991). Genetic covariance of fitness correlates: What genetic correlations are made of and why it matters. Evolution, 45, 630–648. 10.1111/j.1558-5646.1991.tb04334.x 28568816

[ece35932-bib-0030] Kruuk, L. E. B. (2004). Estimating genetic parameters in natural populations using the ‘animal model’. Philosophical Transactions of the Royal Society of London. Series B, Biological Sciences, 359, 873–890. 10.1098/rstb.2003.1437 15306404 PMC1693385

[ece35932-bib-0031] Lande, R. , & Arnold, S. J. (1983). The measurement of selection on correlated characters. Evolution, 37, 1210–1226. 10.1111/j.1558-5646.1983.tb00236.x 28556011

[ece35932-bib-0032] Maynard Smith, J. M. , Burian, R. , Kauffman, S. , Alberch, P. , Campbell, J. , Goodwin, B. , … Wolpert, L. (1985). Developmental constraints and evolution: A perspective from the Mountain Lake conference on development and evolution. The Quarterly Review of Biology, 60, 265–287. 10.1086/414425

[ece35932-bib-0033] Mitchell‐Olds, T. (1996). Pleiotropy causes long‐term genetic constraints on life‐history evolution in *Brassica rapa* . Evolution, 50, 1849–1858. 10.1111/j.1558-5646.1996.tb03571.x 28565577

[ece35932-bib-0034] Morrissey, M. B. , & Wilson, A. J. (2010). pedantics: An R package for pedigree‐based genetic simulation, and pedigree manipulation, characterization, and viewing. Molecular Ecology Resources, 10, 711–719. 10.1111/j.1755-0998.2009.02817.x 21565076

[ece35932-bib-0035] Morton, J. K. (2005) Silene In Flora of North America Editorial Committee (Ed.), Flora of North America (Vol. 5, pp 191–192). New York City: Oxford University Press.

[ece35932-bib-0036] Pavlicev, M. , Kenney‐Hunt, J. P. , Norgard, E. A. , Roseman, C. C. , Wolf, J. B. , & Cheverud, J. M. (2008). Genetic variation in pleiotropy: Differential epistasis as a source of variation in the allometric relationship between long bone lengths and body weight. Evolution, 62, 199–213. 10.1111/j.1558-5646.2007.00255.x 18005158

[ece35932-bib-0037] Revell, L. J. (2012). phytools: An R package for phylogenetic comparative biology (and other things). Methods in Ecology and Evolution, 2, 217–223. 10.1111/j.2041-210X.2011.00169.x

[ece35932-bib-0038] Shykoff, J. A. , & Bucheli, E. (1995). Pollinator visitation patterns, floral rewards and the probability of transmission of *Microbotryum violaceum*, a venereal disease of plants. Journal of Ecology, 83, 189–198. 10.2307/2261557

[ece35932-bib-0039] Sinervo, B. , & Svensson, E. (2002). Correlational selection and the evolution of genomic architecture. Heredity, 89, 329–338. 10.1038/sj.hdy.6800148 12399990

[ece35932-bib-0040] Steven, J. C. , Delph, L. F. , & Brodie, E. D. III (2007). Sexual dimorphism in the quantitative‐genetic architecture of floral, leaf, and allocation traits in *Silene latifolia* . Evolution, 61, 42–57. 10.1111/j.1558-5646.2007.00004.x 17300426

[ece35932-bib-0041] Stinchcombe, J. R. , Weinig, C. , Heath, K. D. , Brock, M. T. , & Schmitt, J. (2009). Polymorphic genes of major effect: Consequences for variation, selection, and evolution in *Arabidopsis thaliana* . Genetics, 182, 911–922. 10.1534/genetics.108.097030 19416942 PMC2710169

[ece35932-bib-0042] Wagner, G. P. , Pavlicev, M. , & Cheverud, J. M. (2007). The road to modularity. Nature Reviews Genetics, 8, 921–931. 10.1038/nrg2267 18007649

[ece35932-bib-0043] Wilson, A. J. , Réale, D. , Clements, M. N. , Morrissey, M. M. , Postma, E. , Walling, C. A. , … Nussey, D. H. (2010). An ecologist's guide to the animal model. Journal of Animal Ecology, 79, 13–26. 10.1111/j.365-2656.2009.01639.x 20409158

